# 7-Bromo-2-(4-methyl­phen­yl)-1-(methyl­sulfin­yl)naphtho­[2,1-*b*]furan

**DOI:** 10.1107/S1600536813016978

**Published:** 2013-06-22

**Authors:** Hong Dae Choi, Pil Ja Seo, Uk Lee

**Affiliations:** a Department of Chemistry, Dongeui University, San 24 Kaya-dong, Busanjin-gu, Busan 614-714, Republic of Korea; b Department of Chemistry, Pukyong National University, 599-1 Daeyeon 3-dong, Nam-gu, Busan 608-737, Republic of Korea

## Abstract

In the title compound, C_20_H_15_BrO_2_S, the dihedral angle between the mean plane [r.m.s. deviation = 0.030 (2) Å] of the naphtho­furan ring system and the 4-methyl­phenyl ring is 38.49 (9)°. In the crystal, mol­ecules are linked by C—H⋯π and C—Br⋯π [3.871 (2) Å] inter­actions into stacks along the *b*-axis direction. These stacks are further linked by weak C—H⋯O hydrogen bonds, forming a three-dimensional network.

## Related literature

For background information and the crystal structures of related compounds, see: Choi *et al.* (2009 ▶, 2010 ▶).
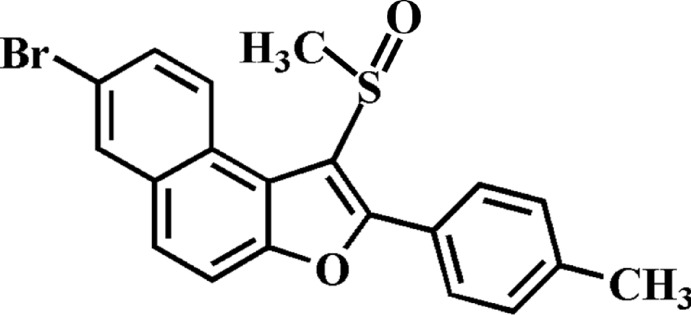


## Experimental

### 

#### Crystal data

C_20_H_15_BrO_2_S
*M* *_r_* = 399.29
Monoclinic, 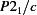

*a* = 6.2198 (11) Å
*b* = 23.234 (4) Å
*c* = 11.344 (2) Å
β = 94.733 (12)°
*V* = 1633.7 (5) Å^3^
*Z* = 4
Mo *K*α radiation
μ = 2.65 mm^−1^
*T* = 173 K
0.27 × 0.19 × 0.11 mm

#### Data collection

Bruker SMART APEXII CCD diffractometer
Absorption correction: multi-scan (*SADABS*; Bruker, 2009 ▶) *T*_min_ = 0.472, *T*_max_ = 0.746
16182 measured reflections
4083 independent reflections
3135 reflections with *I* > 2σ(*I*)
*R*_int_ = 0.070

#### Refinement

*R*[*F* ^2^ > 2σ(*F* ^2^)] = 0.043
*wR*(*F* ^2^) = 0.117
*S* = 1.04
4083 reflections
219 parameters
H-atom parameters constrained
Δρ_max_ = 1.16 e Å^−3^
Δρ_min_ = −0.63 e Å^−3^

### 

Data collection: *APEX2* (Bruker, 2009 ▶); cell refinement: *SAINT* (Bruker, 2009 ▶); data reduction: *SAINT*; program(s) used to solve structure: *SHELXS97* (Sheldrick, 2008 ▶); program(s) used to refine structure: *SHELXL97* (Sheldrick, 2008 ▶); molecular graphics: *ORTEP-3* (Farrugia, 2012 ▶) and *DIAMOND* (Brandenburg, 1998 ▶); software used to prepare material for publication: *SHELXL97*.

## Supplementary Material

Crystal structure: contains datablock(s) global, I. DOI: 10.1107/S1600536813016978/hg5324sup1.cif

Structure factors: contains datablock(s) I. DOI: 10.1107/S1600536813016978/hg5324Isup2.hkl

Supplementary material file. DOI: 10.1107/S1600536813016978/hg5324Isup3.cml

Additional supplementary materials:  crystallographic information; 3D view; checkCIF report

## Figures and Tables

**Table 1:** Hydrogen-bond geometry (Å, °) *Cg*1 is the centroid of the C2/C3/C8/C9/C10/C11 benzene ring.

*D*—H⋯*A*	*D*—H	H⋯*A*	*D*⋯*A*	*D*—H⋯*A*
C19—H19*A*⋯O2^i^	0.98	2.52	3.476 (4)	166
C14—H14⋯*Cg*1^ii^	0.95	2.68	3.342 (4)	127

Symmetry codes: (i) 
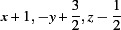
; (ii) 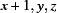.

**Fig. 1.**
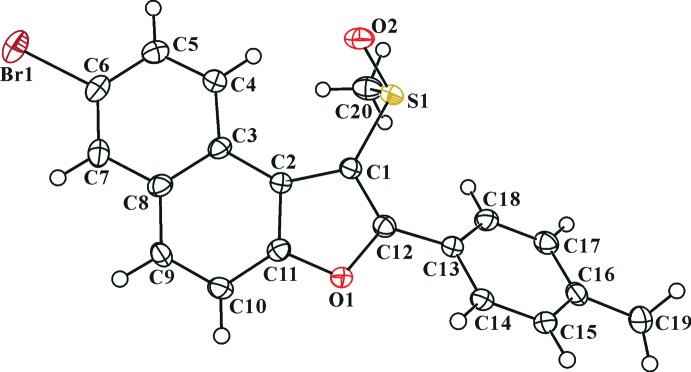
The molecular structure of the title compound with the atom numbering scheme. Displacement ellipsoids are drawn at the 50% probability level. H atoms are presented as small spheres of arbitrary radius.

**Fig. 2.**
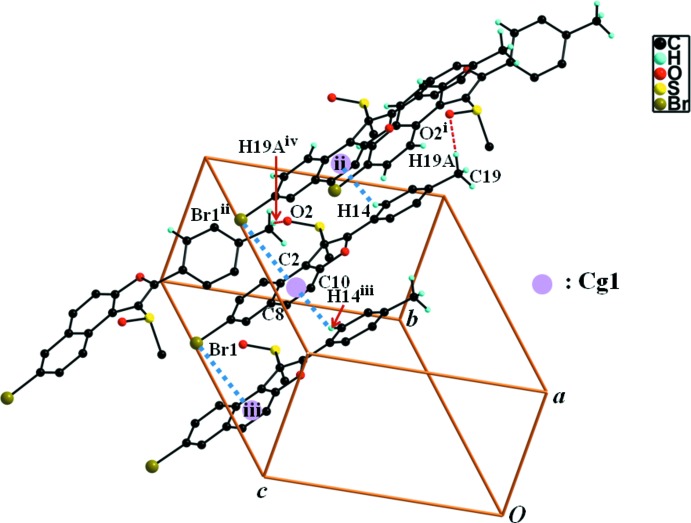
A view of the C–H···O, C–H···π and C–Br···π interactions (dotted lines) in the crystal structure of the title compound. H atoms non-participating in hydrogen-bonding were omitted for clarity. [Symmetry codes: (i) x + 1, - y + 3/2, z - 1/2; (ii) x+ 1, y, z; (iii) x - 1, y, z; (iv) x - 1, - y + 3/2, z + 1/2.]

## supplementary crystallographic information

### Comment

As a part of our ongoing study of 7-bromo-1-(methylsulfinyl)naphtho[2,1-b] furan derivatives containing phenyl (Choi *et al.*, 2009) and 4-fluorophenyl (Choi *et al.*, 2010) substituents in 2-position, we report herein the crystal structure of the title compound.

In the title molecule (Fig. 1), the naphthofuran unit is essentially planar, with a mean deviation of 0.030 (2) Å from the least-squares plane defined by the thirteen constituent atoms. The dihedral angle between the mean plane of the naphthofuran ring system and the 4-methylphenyl ring is 38.49 (9)°. In the crystal structure (Fig. 2), molecules are connected by C–H···π (Table 1), and C6–Br1···π [3.871 (2) Å] interactions between the bromine atom and the central benzene ring of a neighbouring molecule with a Br1···Cg1^iii^ being 3.871 (2) Å (Cg1 is the centroid of the C2/C3/C8/C9/C10/C11 benzene ring), into stacks along the *b-axis* direction. These stacks are further packed by weak C–H···O hydrogen bonds (Table 1), forming a three-dimensional network.

### Experimental

3-Chloroperoxybenzoic acid (77%, 202 mg, 0.9 mmol) was added in small portions to a stirred solution of 7-bromo-2-(4-methylphenyl)-1-(methylsulfanyl)naphtho[2,1-b]furan (306 mg, 0.8 mmol) in dichloromethane (40 mL) at 273 K. After being stirred at room temperature for 6h, the mixture was washed with saturated sodium bicarbonate solution and the organic layer was separated, dried over magnesium sulfate, filtered and concentrated at reduced pressure. The residue was purified by column chromatography (hexane-ethyl acetate, 2:1 v/v) to afford the title compound as a colorless solid [yield 61%, m.p. 485-486 K; *R*_f_ = 0.46 (hexane-ethyl acetate, 2:1 v/v)]. Single crystals suitable for X-ray diffraction were prepared by slow evaporation of a solution of the title compound in acetone at room temperature.

### Refinement

All H atoms were positioned geometrically and refined using a riding model, with C–H = 0.95 Å for aryl and 0.98Å for methyl H atoms. *U*_iso_(H) = 1.2*U*_eq_(C) for aryl and 1.5*U*_eq_(C) for methyl H atoms. The positions of methyl hydrogens were optimized rotationally. The highest peak in the difference map is 1.38 Å from S1 and the largest hole is 0.70 Å from Br1.

### Crystal data

**Table:**

C_20_H_15_BrO_2_S	*F*(000) = 808
*M**_r_* = 399.29	*D*_x_ = 1.623 Mg m^−^^3^
Monoclinic, *P*2_1_/*c*	Melting point = 485–486 K
Hall symbol: -P 2ybc	Mo *K*α radiation, λ = 0.71073 Å
*a* = 6.2198 (11) Å	Cell parameters from 3620 reflections
*b* = 23.234 (4) Å	θ = 2.5–27.0°
*c* = 11.344 (2) Å	µ = 2.65 mm^−^^1^
β = 94.733 (12)°	*T* = 173 K
*V* = 1633.7 (5) Å^3^	Block, colourless
*Z* = 4	0.27 × 0.19 × 0.11 mm

### Data collection

**Table:**

Bruker SMART APEXII CCD diffractometer	4083 independent reflections
Radiation source: rotating anode	3135 reflections with *I* > 2σ(*I*)
Graphite multilayer monochromator	*R*_int_ = 0.070
Detector resolution: 10.0 pixels mm^-1^	θ_max_ = 28.4°, θ_min_ = 1.8°
φ and ω scans	*h* = −8→8
Absorption correction: multi-scan (*SADABS*; Bruker, 2009)	*k* = −30→30
*T*_min_ = 0.472, *T*_max_ = 0.746	*l* = −15→15
16182 measured reflections	

### Refinement

**Table:**

Refinement on *F*^2^	Primary atom site location: structure-invariant direct methods
Least-squares matrix: full	Secondary atom site location: difference Fourier map
*R*[*F*^2^ > 2σ(*F*^2^)] = 0.043	Hydrogen site location: difference Fourier map
*wR*(*F*^2^) = 0.117	H-atom parameters constrained
*S* = 1.04	*w* = 1/[σ^2^(*F*_o_^2^) + (0.0557*P*)^2^ + 0.6314*P*] where *P* = (*F*_o_^2^ + 2*F*_c_^2^)/3
4083 reflections	(Δ/σ)_max_ = 0.001
219 parameters	Δρ_max_ = 1.16 e Å^−^^3^
0 restraints	Δρ_min_ = −0.63 e Å^−^^3^

### Special details

**Table:**

Geometry. All esds (except the esd in the dihedral angle between two l.s. planes) are estimated using the full covariance matrix. The cell esds are taken into account individually in the estimation of esds in distances, angles and torsion angles; correlations between esds in cell parameters are only used when they are defined by crystal symmetry. An approximate (isotropic) treatment of cell esds is used for estimating esds involving l.s. planes.
Refinement. Refinement of F^2^ against ALL reflections. The weighted R-factor wR and goodness of fit S are based on F^2^, conventional R-factors R are based on F, with F set to zero for negative F^2^. The threshold expression of F^2^ > 2sigma(F^2^) is used only for calculating R-factors(gt) etc. and is not relevant to the choice of reflections for refinement. R-factors based on F^2^ are statistically about twice as large as those based on F, and R- factors based on ALL data will be even larger.

### Fractional atomic coordinates and isotropic or equivalent isotropic displacement parameters (Å^2^)

**Table:**

	*x*	*y*	*z*	*U*_iso_*/*U*_eq_	
Br1	0.08040 (5)	0.625454 (16)	1.02806 (3)	0.03661 (13)	
S1	0.99799 (11)	0.71743 (3)	0.64298 (6)	0.02279 (16)	
O1	1.0693 (3)	0.54998 (8)	0.62255 (17)	0.0226 (4)	
O2	0.9682 (3)	0.74231 (8)	0.76168 (19)	0.0299 (5)	
C1	0.9742 (4)	0.64212 (12)	0.6514 (2)	0.0198 (5)	
C2	0.8386 (4)	0.60547 (11)	0.7188 (2)	0.0198 (5)	
C3	0.6611 (4)	0.61296 (12)	0.7902 (2)	0.0216 (6)	
C4	0.5747 (4)	0.66676 (12)	0.8204 (2)	0.0235 (6)	
H4	0.6369	0.7011	0.7932	0.028*	
C5	0.4029 (5)	0.67024 (13)	0.8883 (3)	0.0267 (6)	
H5	0.3461	0.7067	0.9075	0.032*	
C6	0.3116 (4)	0.61977 (13)	0.9291 (3)	0.0259 (6)	
C7	0.3880 (4)	0.56710 (13)	0.9027 (2)	0.0264 (6)	
H7	0.3220	0.5335	0.9310	0.032*	
C8	0.5663 (4)	0.56194 (12)	0.8330 (2)	0.0236 (6)	
C9	0.6476 (4)	0.50695 (12)	0.8070 (2)	0.0253 (6)	
H9	0.5821	0.4738	0.8374	0.030*	
C10	0.8171 (4)	0.50023 (12)	0.7395 (2)	0.0253 (6)	
H10	0.8715	0.4632	0.7222	0.030*	
C11	0.9068 (4)	0.55038 (12)	0.6971 (2)	0.0213 (5)	
C12	1.1077 (4)	0.60658 (12)	0.5948 (2)	0.0207 (5)	
C13	1.2659 (4)	0.61466 (11)	0.5080 (2)	0.0206 (5)	
C14	1.4444 (4)	0.57774 (12)	0.5083 (2)	0.0214 (5)	
H14	1.4675	0.5495	0.5685	0.026*	
C15	1.5871 (4)	0.58234 (12)	0.4214 (3)	0.0231 (6)	
H15	1.7088	0.5575	0.4235	0.028*	
C16	1.5555 (5)	0.62264 (12)	0.3308 (3)	0.0239 (6)	
C17	1.3798 (4)	0.65974 (12)	0.3322 (2)	0.0252 (6)	
H17	1.3581	0.6883	0.2725	0.030*	
C18	1.2359 (4)	0.65572 (12)	0.4190 (2)	0.0239 (6)	
H18	1.1162	0.6812	0.4176	0.029*	
C19	1.7039 (5)	0.62599 (13)	0.2328 (3)	0.0309 (7)	
H19A	1.8006	0.6591	0.2460	0.046*	
H19B	1.6183	0.6305	0.1569	0.046*	
H19C	1.7894	0.5906	0.2316	0.046*	
C20	0.7540 (5)	0.73215 (13)	0.5513 (3)	0.0318 (7)	
H20A	0.6305	0.7158	0.5881	0.048*	
H20B	0.7635	0.7148	0.4732	0.048*	
H20C	0.7350	0.7739	0.5428	0.048*	

### Atomic displacement parameters (Å^2^)

**Table:**

	*U*^11^	*U*^22^	*U*^33^	*U*^12^	*U*^13^	*U*^23^
Br1	0.03055 (19)	0.0514 (2)	0.02954 (18)	0.00701 (13)	0.01237 (13)	0.00111 (14)
S1	0.0252 (3)	0.0159 (3)	0.0275 (4)	−0.0011 (2)	0.0034 (3)	−0.0017 (3)
O1	0.0257 (9)	0.0171 (10)	0.0262 (10)	0.0019 (7)	0.0083 (8)	0.0005 (8)
O2	0.0372 (11)	0.0225 (11)	0.0302 (11)	0.0008 (9)	0.0029 (9)	−0.0079 (9)
C1	0.0216 (13)	0.0176 (13)	0.0203 (12)	−0.0008 (10)	0.0021 (10)	−0.0009 (10)
C2	0.0215 (13)	0.0179 (13)	0.0202 (13)	−0.0009 (10)	0.0032 (10)	−0.0013 (10)
C3	0.0237 (13)	0.0229 (14)	0.0183 (12)	0.0007 (10)	0.0032 (10)	−0.0037 (11)
C4	0.0249 (14)	0.0213 (14)	0.0245 (14)	−0.0017 (11)	0.0025 (11)	−0.0016 (11)
C5	0.0262 (14)	0.0259 (16)	0.0282 (15)	0.0042 (11)	0.0027 (11)	−0.0040 (12)
C6	0.0215 (13)	0.0361 (17)	0.0204 (13)	0.0013 (11)	0.0035 (11)	−0.0035 (12)
C7	0.0256 (14)	0.0316 (16)	0.0226 (14)	−0.0043 (12)	0.0060 (11)	0.0021 (12)
C8	0.0284 (14)	0.0237 (15)	0.0189 (13)	0.0015 (11)	0.0027 (10)	−0.0032 (11)
C9	0.0316 (15)	0.0193 (14)	0.0257 (14)	−0.0062 (11)	0.0072 (11)	0.0017 (11)
C10	0.0323 (15)	0.0174 (14)	0.0267 (14)	0.0019 (11)	0.0048 (12)	0.0010 (11)
C11	0.0215 (12)	0.0215 (14)	0.0209 (13)	0.0026 (10)	0.0022 (10)	−0.0002 (11)
C12	0.0224 (13)	0.0199 (14)	0.0197 (13)	−0.0007 (10)	0.0009 (10)	−0.0003 (10)
C13	0.0222 (13)	0.0181 (13)	0.0220 (13)	−0.0016 (10)	0.0039 (10)	−0.0022 (10)
C14	0.0242 (13)	0.0172 (13)	0.0232 (13)	0.0003 (10)	0.0034 (10)	0.0005 (11)
C15	0.0210 (13)	0.0195 (14)	0.0293 (14)	0.0005 (10)	0.0041 (11)	−0.0020 (11)
C16	0.0265 (14)	0.0202 (14)	0.0254 (14)	−0.0060 (11)	0.0050 (11)	−0.0006 (11)
C17	0.0316 (15)	0.0204 (14)	0.0238 (14)	−0.0003 (11)	0.0028 (11)	0.0038 (11)
C18	0.0264 (14)	0.0198 (14)	0.0256 (14)	0.0027 (11)	0.0036 (11)	0.0009 (11)
C19	0.0328 (16)	0.0291 (17)	0.0320 (16)	−0.0043 (12)	0.0103 (13)	0.0023 (13)
C20	0.0398 (17)	0.0244 (16)	0.0302 (16)	0.0060 (13)	−0.0032 (13)	0.0007 (13)

### Geometric parameters (Å, º)

**Table:**

Br1—C6	1.900 (3)	C9—H9	0.9500
S1—O2	1.491 (2)	C10—C11	1.395 (4)
S1—C1	1.759 (3)	C10—H10	0.9500
S1—C20	1.801 (3)	C12—C13	1.460 (4)
O1—C11	1.370 (3)	C13—C18	1.390 (4)
O1—C12	1.378 (3)	C13—C14	1.403 (4)
C1—C12	1.368 (4)	C14—C15	1.384 (4)
C1—C2	1.459 (4)	C14—H14	0.9500
C2—C11	1.377 (4)	C15—C16	1.392 (4)
C2—C3	1.433 (4)	C15—H15	0.9500
C3—C4	1.414 (4)	C16—C17	1.393 (4)
C3—C8	1.427 (4)	C16—C19	1.504 (4)
C4—C5	1.370 (4)	C17—C18	1.388 (4)
C4—H4	0.9500	C17—H17	0.9500
C5—C6	1.399 (4)	C18—H18	0.9500
C5—H5	0.9500	C19—H19A	0.9800
C6—C7	1.355 (4)	C19—H19B	0.9800
C7—C8	1.419 (4)	C19—H19C	0.9800
C7—H7	0.9500	C20—H20A	0.9800
C8—C9	1.414 (4)	C20—H20B	0.9800
C9—C10	1.362 (4)	C20—H20C	0.9800
			
O2—S1—C1	108.63 (13)	O1—C11—C10	122.9 (2)
O2—S1—C20	106.64 (14)	C2—C11—C10	125.3 (3)
C1—S1—C20	98.61 (14)	C1—C12—O1	110.2 (2)
C11—O1—C12	106.6 (2)	C1—C12—C13	135.0 (3)
C12—C1—C2	107.1 (2)	O1—C12—C13	114.6 (2)
C12—C1—S1	121.2 (2)	C18—C13—C14	118.7 (3)
C2—C1—S1	131.6 (2)	C18—C13—C12	121.3 (3)
C11—C2—C3	118.5 (2)	C14—C13—C12	119.8 (2)
C11—C2—C1	104.4 (2)	C15—C14—C13	120.3 (3)
C3—C2—C1	137.0 (3)	C15—C14—H14	119.9
C4—C3—C8	118.4 (2)	C13—C14—H14	119.9
C4—C3—C2	124.8 (3)	C14—C15—C16	121.2 (3)
C8—C3—C2	116.8 (2)	C14—C15—H15	119.4
C5—C4—C3	121.2 (3)	C16—C15—H15	119.4
C5—C4—H4	119.4	C15—C16—C17	118.1 (3)
C3—C4—H4	119.4	C15—C16—C19	121.5 (3)
C4—C5—C6	119.6 (3)	C17—C16—C19	120.4 (3)
C4—C5—H5	120.2	C18—C17—C16	121.3 (3)
C6—C5—H5	120.2	C18—C17—H17	119.4
C7—C6—C5	121.6 (3)	C16—C17—H17	119.4
C7—C6—Br1	119.3 (2)	C17—C18—C13	120.4 (3)
C5—C6—Br1	119.0 (2)	C17—C18—H18	119.8
C6—C7—C8	120.3 (3)	C13—C18—H18	119.8
C6—C7—H7	119.9	C16—C19—H19A	109.5
C8—C7—H7	119.9	C16—C19—H19B	109.5
C9—C8—C7	120.1 (3)	H19A—C19—H19B	109.5
C9—C8—C3	121.0 (2)	C16—C19—H19C	109.5
C7—C8—C3	118.9 (3)	H19A—C19—H19C	109.5
C10—C9—C8	121.8 (3)	H19B—C19—H19C	109.5
C10—C9—H9	119.1	S1—C20—H20A	109.5
C8—C9—H9	119.1	S1—C20—H20B	109.5
C9—C10—C11	116.6 (3)	H20A—C20—H20B	109.5
C9—C10—H10	121.7	S1—C20—H20C	109.5
C11—C10—H10	121.7	H20A—C20—H20C	109.5
O1—C11—C2	111.7 (2)	H20B—C20—H20C	109.5
			
O2—S1—C1—C12	−139.7 (2)	C12—O1—C11—C2	−0.1 (3)
C20—S1—C1—C12	109.4 (2)	C12—O1—C11—C10	176.8 (2)
O2—S1—C1—C2	35.1 (3)	C3—C2—C11—O1	176.7 (2)
C20—S1—C1—C2	−75.8 (3)	C1—C2—C11—O1	−0.5 (3)
C12—C1—C2—C11	0.8 (3)	C3—C2—C11—C10	−0.1 (4)
S1—C1—C2—C11	−174.6 (2)	C1—C2—C11—C10	−177.2 (3)
C12—C1—C2—C3	−175.5 (3)	C9—C10—C11—O1	−176.0 (2)
S1—C1—C2—C3	9.1 (5)	C9—C10—C11—C2	0.5 (4)
C11—C2—C3—C4	179.6 (3)	C2—C1—C12—O1	−0.9 (3)
C1—C2—C3—C4	−4.5 (5)	S1—C1—C12—O1	175.08 (18)
C11—C2—C3—C8	−0.6 (4)	C2—C1—C12—C13	174.0 (3)
C1—C2—C3—C8	175.3 (3)	S1—C1—C12—C13	−10.0 (5)
C8—C3—C4—C5	−0.5 (4)	C11—O1—C12—C1	0.6 (3)
C2—C3—C4—C5	179.3 (3)	C11—O1—C12—C13	−175.4 (2)
C3—C4—C5—C6	0.3 (4)	C1—C12—C13—C18	−35.4 (5)
C4—C5—C6—C7	−0.4 (5)	O1—C12—C13—C18	139.3 (3)
C4—C5—C6—Br1	177.7 (2)	C1—C12—C13—C14	149.3 (3)
C5—C6—C7—C8	0.6 (4)	O1—C12—C13—C14	−36.0 (3)
Br1—C6—C7—C8	−177.4 (2)	C18—C13—C14—C15	−0.1 (4)
C6—C7—C8—C9	179.1 (3)	C12—C13—C14—C15	175.4 (3)
C6—C7—C8—C3	−0.8 (4)	C13—C14—C15—C16	−1.2 (4)
C4—C3—C8—C9	−179.2 (2)	C14—C15—C16—C17	2.2 (4)
C2—C3—C8—C9	1.0 (4)	C14—C15—C16—C19	−177.0 (3)
C4—C3—C8—C7	0.8 (4)	C15—C16—C17—C18	−2.0 (4)
C2—C3—C8—C7	−179.1 (2)	C19—C16—C17—C18	177.2 (3)
C7—C8—C9—C10	179.4 (3)	C16—C17—C18—C13	0.8 (4)
C3—C8—C9—C10	−0.7 (4)	C14—C13—C18—C17	0.3 (4)
C8—C9—C10—C11	−0.1 (4)	C12—C13—C18—C17	−175.1 (3)

### Hydrogen-bond geometry (Å, º)

Cg1 is the centroid of the C2/C3/C8/C9/C10/C11 benzene ring.

**Table:**

*D*—H···*A*	*D*—H	H···*A*	*D*···*A*	*D*—H···*A*
C19—H19*A*···O2^i^	0.98	2.52	3.476 (4)	166
C14—H14···*Cg*1^ii^	0.95	2.68	3.342 (4)	127

Symmetry codes: (i) *x*+1, −*y*+3/2, *z*−1/2; (ii) *x*+1, *y*, *z*.
